# Iron homeostasis disruption and lipid peroxidation in skeletal muscle during short‐term immobilization

**DOI:** 10.1002/2211-5463.70223

**Published:** 2026-03-19

**Authors:** Haruka Yokogawa, Kazuhiko Higashida, Naoya Nakai

**Affiliations:** ^1^ Laboratory of Exercise Nutrition, Department of Nutrition University of Shiga Prefecture Hikone Japan

**Keywords:** atrophy, casting, iron homeostasis, muscle disuse, transferrin receptor

## Abstract

Disuse‐induced muscle atrophy may involve dysregulated iron metabolism, yet the underlying mechanisms are unclear. We examined iron homeostasis in mouse gastrocnemius muscle following 14‐day casting‐induced immobilization. Muscle mass declined by ~ 25%, accompanied by increased nonheme iron and ferritin heavy chain levels. Despite iron accumulation, transferrin receptor 1 and iron regulatory protein 2 were paradoxically upregulated, suggesting disruption of IRP/IRE feedback control. Additionally, levels of 4‐hydroxynonenal, a marker of lipid peroxidation, were elevated without compensatory responses from SLC7A11/xCT or GPx4. These findings indicate that short‐term immobilization disrupts iron regulatory mechanisms and induces oxidative stress exceeding antioxidant capacity. Iron dysregulation and lipid peroxidation may contribute to the early pathogenesis of disuse‐induced muscle atrophy and represent potential therapeutic targets.

## Graphical abstract

14‐day casting‐induced immobilization reduced gastrocnemius muscle mass and increased non‐heme iron and ferritin heavy chain levels. Despite iron accumulation, transferrin receptor 1 and iron regulatory protein 2 were paradoxically upregulated. Lipid peroxidation was elevated without compensatory antioxidant responses. Iron dysregulation and lipid peroxidation may contribute to the early pathogenesis of disuse‐induced muscle atrophy and represent potential therapeutic targets.

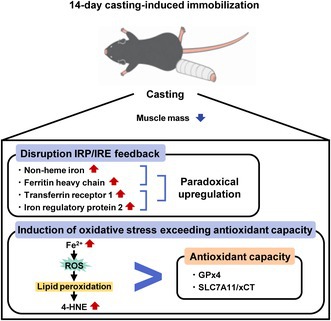

**Abbreviations**
4‐HNE: 4‐hydroxynonenal
CBB: Coomassie Brilliant Blue
DMT1: divalent metal transporter 1
fpn: ferroportin
FTH1: ferritin heavy chain 1
FTL: ferritin light chain
GPx4: glutathione peroxidase 4
IRP/IRE: iron regulatory protein/iron‐responsive element
IRP2: iron regulatory protein 2
MuRF1: muscle RING‐finger 1
ROS: reactive oxygen species
SOD: superoxide dismutase
TfR1: transferrin receptor 1

Loss of skeletal muscle mass is a well‐documented consequence of aging and is a major risk factor for frailty, metabolic dysfunction, and diminished quality of life in older adults [1]. Sarcopenia is an age‐related decline in muscle quantity and function, driven by a chronic imbalance between muscle protein synthesis and degradation. Understanding the molecular mechanisms underlying muscle atrophy is essential for developing strategies for preserving muscle mass even while aging.

Among the many factors that influence muscle health, iron plays a paradoxical role. While it is required for critical cellular processes, such as oxygen transport, mitochondrial respiration, and DNA synthesis, excess iron is biologically hazardous due to its ability to catalyze the formation of reactive oxygen species (ROS) via the Fenton reaction. The ROS, hence produced, can damage cellular structures and contribute to the accumulation of lipid peroxides, which can result in the recently characterized form of regulated cell death known as ferroptosis [2]. Ferroptosis has been implicated in various pathological processes, including neurodegeneration and ischemia–reperfusion injury [3]. Recent studies suggest that iron accumulation, with aging, may promote sarcopenia [4, 5], thereby raising questions about the broad relevance of iron dysregulation in muscle‐wasting conditions.

Although the role of iron in age‐related muscle loss is increasingly recognized, whether similar iron‐related mechanisms contribute to muscle atrophy in other contexts, such as disuse, still remains unclear. Disuse‐induced muscle atrophy, triggered by mechanical unloading or physical inactivity, is frequently encountered in clinical and spaceflight settings and is characterized by the rapid loss of muscle mass and strength [6, 7]. Although the molecular signaling pathways involved in protein degradation during disuse have been extensively studied [8, 9], the effect of immobilization on iron homeostasis and whether iron overload could contribute to disuse‐induced oxidative damage remains unknown.

In this study, we investigated whether short‐term disuse, induced by casting immobilization, could alter iron metabolism in skeletal muscles. Specifically, we assessed nonheme iron accumulation, expression of iron transport and regulatory proteins, the markers of lipid peroxidation, and antioxidant capacity in the gastrocnemius muscle of mice. In this study, we aimed to determine whether iron dysregulation could play a role in the early pathogenesis of muscle disuse atrophy, independent of aging‐related mechanisms.

## Materials and methods

### Animal care

All reagents were obtained from Nacalai Tesque (Kyoto, Japan) unless indicated otherwise. The experimental protocol was approved by the Animal Experimental Committee of the University of Shiga Prefecture (approval no. 2024‐2). Seven‐week‐old male C57BL/6J mice were purchased from CLEA (Tokyo, Japan). The animals were maintained under a constant light/dark cycle (light from 6 am to 6 pm) and kept in plastic cages with bedding material. All mice were provided free access to food (CE‐2; CLEA Japan, Tokyo, Japan), containing 28.9 mg/100 g diet and tap water throughout the experiment. After a 1‐week acclimation period, unilateral hindlimb immobilization was performed on the right hindlimb, as described previously [10, 11, 12]. Briefly, the mice were anesthetized with isoflurane for attachment of the casting material. The right hind limb was fixed in a shortened position with complete plantar flexion of the ankle joint using a casting tape (Scotchcast Plus‐J; 3 M Health Care, St. Paul, MN, USA). The animals were checked daily for damage to the casting material, which was repaired as and when required. The mice were then immobilized for 14 days. After 14‐day immobilization, they were euthanized by cervical dislocation, and the gastrocnemius muscle was harvested.

### Western blotting

The gastrocnemius muscle was homogenized in ice‐cold lysis buffer containing 50 mm Tris–HCl (pH 7.4), 150 mm NaCl, 0.25% deoxycholic acid, 1% NP‐40, 1 mm EDTA, and a protease/phosphatase inhibitor cocktail. The homogenates were centrifuged at 15 000× ***g*** for 5 min at 4 °C. The supernatants were collected, and protein concentrations were measured using a bicinchoninic acid assay kit. Samples were prepared in 4× Laemmli sample buffer. Twenty to 40 μg of proteins were separated by 7.5%, 10%, or 15% SDS/PAGE, transferred to a PVDF membrane, and incubated overnight at 4 °C with primary antibodies. Anti‐ferritin heavy chain 1 (FTH1) monoclonal antibody (#4393), anti‐glutathione peroxidase (GPx) 4 polyclonal antibody (#52455), anti‐superoxide dismutase (SOD) 2 monoclonal antibody (#13141), and all secondary antibodies were purchased from Cell Signaling Technology (Danvers, MA, USA). Anti‐cytochrome c monoclonal antibody (sc‐13156), anti‐MuRF1 monoclonal antibody (sc‐398 608), and anti‐myoglobin monoclonal antibody (sc‐74525) were purchased from Santa Cruz Biotechnology (Dallas, TX, USA). Anti‐transferrin receptor (TfR) 1 monoclonal antibody (13‐6800) was purchased from Thermo Fisher Scientific (Waltham, MA, USA). Anti‐ferroportin (fpn) polyclonal antibody (NBP 1‐21502) was obtained from Novus Biologicals (Centennial, CO, USA). Anti‐ferritin light chain (FTL) polyclonal antibody (10727‐1‐AP) was purchased from Proteintech (Rosemont, IL, USA). The anti‐iron regulatory protein (IRP) 2 monoclonal antibody (MABS2030) was obtained from EMD Millipore (Burlington, MA, USA). Anti‐4‐hydroxynonenal (4‐HNE) polyclonal antibody (ab46545) and anti‐SOD1 polyclonal antibody (ab62800) were purchased from Abcam (Cambridge, UK). Anti‐SOD3 polyclonal antibody (AF4817) was purchased from R&D systems (Minneapolis, MN, USA). Bands were visualized using enhanced chemiluminescence (Merck, Darmstadt, Germany) and quantified using densitometry. Equal loading was confirmed by staining the blot with Coomassie Brilliant Blue (CBB) [13].

### RNA extraction and real‐time PCR

The gastrocnemius muscle was homogenized in Isogen (NIPPON GENE, Tokyo, Japan), and total RNA was extracted and precipitated using chloroform and isopropanol. DNase‐treated total RNA (2 μg) was reverse‐transcribed into cDNA using a random primer and High‐Capacity cDNA Reverse Transcription Kit (Applied Biosystems, Foster City, CA, USA). Real‐time PCR was performed using a QuantStudio Real‐Time PCR System (Applied Biosystems, Foster City, CA, USA). Gene expression was determined using commercially available Taqman primers and probes for divalent metal transporter 1 (DMT‐1), SLC7A11/xCT, β‐actin, and Taqman Fast Advanced Master Mix (Applied Biosystems, Foster City, CA, USA). The levels of gene expression were determined using the ΔΔ*C*_T_ method, using β‐actin as the housekeeping gene.

### Nonheme iron content

Nonheme iron content of gastrocnemius muscle was measured by a slight modification of the method described by Torrence and Bothwell [14]. Briefly, gastrocnemius muscles were homogenized in ice‐cold 0.5 m HCl and 5% trichloroacetic acid. The homogenates were boiled at 100 °C for 30 min and centrifuged at 15 000× ***g*** for 10 min. Aliquots of the supernatants were used to measure nonheme iron content by bathophenanthroline method (L‐type Wako FeN, Fujifilm, Tokyo, Japan).

### Statistical analysis

Data are expressed as means ± standard deviation (SD). Statistical analyses were performed using the Bell Curve for Excel (Social Survey Research Information, Tokyo, Japan). Differences between groups were assessed using paired t‐tests, as indicated. Differences were considered statistically significant at *P* < 0.05.

## Results

### Muscle atrophy and nonheme iron accumulation

**Fig. 1:**
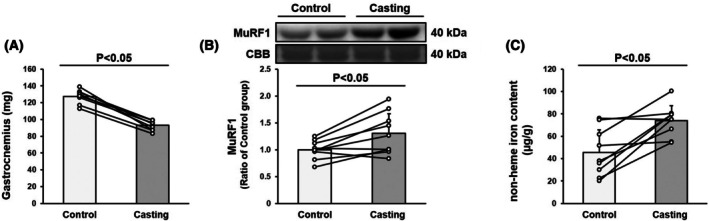
Effects of 2‐week casting immobilization on skeletal muscle weight, MuRF1 expression, and nonheme iron content. (A) Gastrocnemius muscle mass. (B) Representative blots and quantitation of MuRF1. (C) Nonheme iron content in the gastrocnemius muscle; *n* = 9. Data are shown as the mean ± SD. Paired *t*‐test was used for statistical analysis.

We first investigated skeletal muscle atrophy and the accumulation of nonheme iron following cast immobilization. As shown in Fig. 1, 14 days of immobilization led to an approximately 25% reduction in the gastrocnemius muscle mass (Fig. 1A). To confirm the presence of disuse‐induced muscle atrophy, we examined the expression of muscle RING‐finger 1 (MuRF1), a ubiquitin ligase that serves as a well‐established catabolic marker of skeletal muscle atrophy. MuRF1 expression was significantly increased in the immobilized muscle (Fig. 1B). Moreover, nonheme iron content was significantly elevated in the immobilized muscle (Fig. 1C).

### Ferritin and heme‐associated proteins

**Fig. 2:**
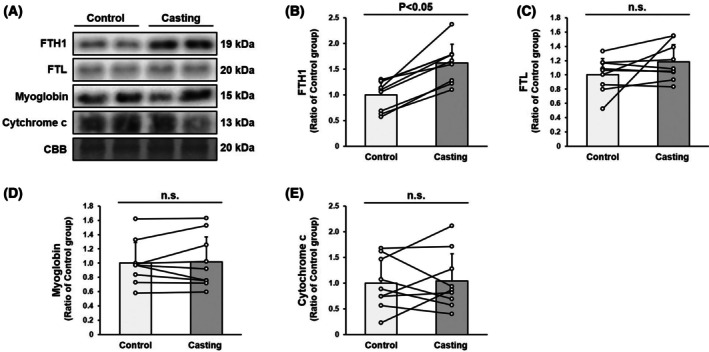
Effects of 2‐week casting immobilization on iron‐related proteins in skeletal muscle. (A) Representative blots for and quantitation of (B) FTH1, (C) FTL, (D) myoglobin, and (E) cytochrome c; *n* = 9. Data are shown as the mean ± SD. Paired *t*‐test was used for statistical analysis.

Intracellular iron is primarily stored in ferritin, which is composed of heavy and light chains. Since ferritin expression increases in response to elevated intracellular iron levels, we analyzed ferritin levels in the gastrocnemius muscle. Fig. 2 shows that ferritin heavy chain 1 (FTH1) expression was significantly upregulated in atrophied muscles, whereas ferritin light chain (FTL) expression remained unchanged (Fig. 2B,C). Nonheme iron serves as a precursor for mitochondrial heme biosynthesis. Therefore, its accumulation might reflect impaired heme production. To test this hypothesis, we assessed the expression of myoglobin and cytochrome c, which are representative heme‐containing proteins. No significant change was observed in their expression following immobilization, suggesting that the increased nonheme iron levels were not due to impaired heme synthesis.

### Iron uptake and regulatory proteins

**Fig. 3:**
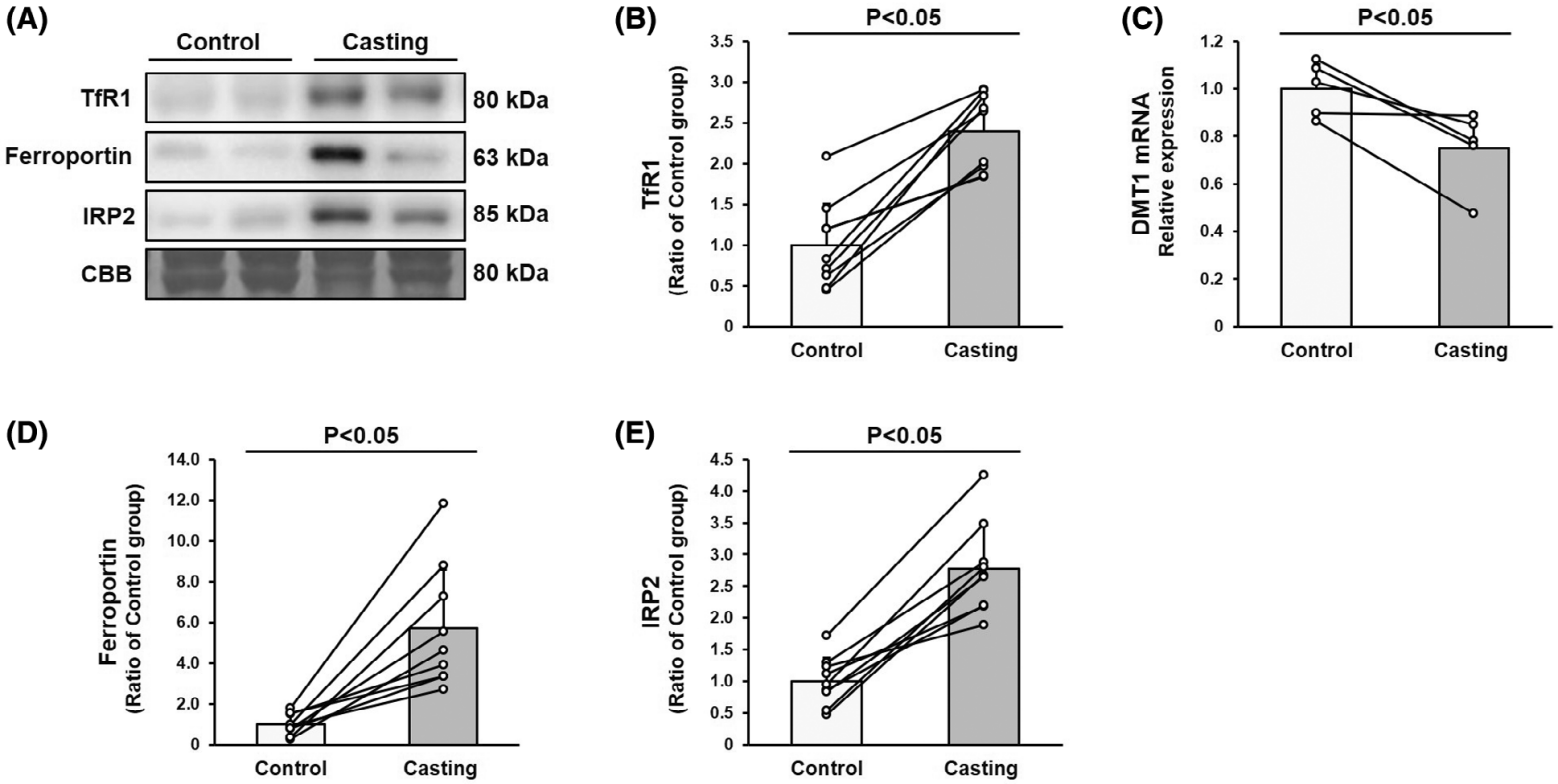
Effects of 2‐week casting immobilization on iron transport proteins in skeletal muscle. (A) Representative blots for TfR1, Fpn, and IRP2. (B) Quantitation of TfR1; *n* = 9. (C) Expression of DMT1 mRNA; *n* = 5. Quantification of (D) Fpn and (E) IRP2; *n* = 9. Data are shown as the mean ± SD. Paired *t*‐test was used for statistical analysis.

To elucidate the mechanism underlying nonheme iron accumulation in casting‐induced atrophy, we analyzed the expression of iron‐uptake proteins. Transferrin receptor 1 (TfR1), which facilitates cellular iron import from transferrin‐bound iron, was significantly upregulated in the immobilized muscles (Fig. 3B). Due to the lack of reliable antibodies, DMT1 expression was assessed by RT‐PCR, which revealed a slight decrease in its expression (Fig. 3C). Ferroportin, the only known mammalian iron exporter, also showed an elevated expression following immobilization (Fig. 3D). Under iron‐replete conditions, TfR1 and iron regulatory protein 2 (IRP2) are typically downregulated to prevent iron overload. However, IRP2 expression was remarkably increased in the immobilized muscle (Fig. 3E). The findings together suggested that casting‐induced muscle atrophy disrupted iron homeostasis in skeletal muscle.

### Antioxidant system response

**Fig. 4:**
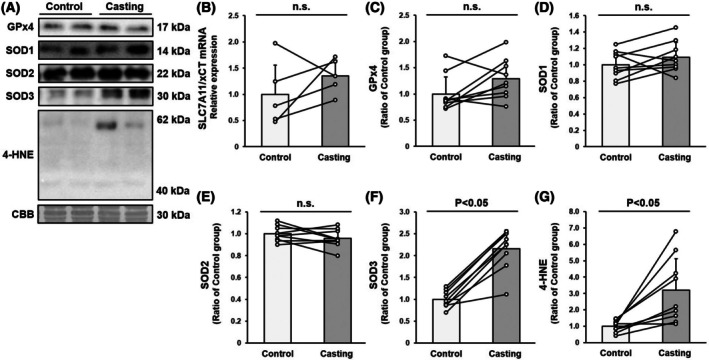
Effects of 2‐week casting immobilization on antioxidant proteins and 4‐HNE in skeletal muscle. (A) Representative blots for antioxidant proteins and 4‐HNE. (B) Expression level of xCT mRNA; *n* = 5. Quantitation of (C) GPx4, (D) SOD1, (E) SOD2, (F) SOD3 and (G) 4‐HNE; *n* = 9. Data are shown as the mean ± SD. Paired *t*‐test was used for statistical analysis.

Cells possess intrinsic antioxidant systems that counteract the oxidative stress caused by iron overload. SLC7A11/xCT is a cystine/glutamate antiporter that promotes cystine uptake, which is necessary for intracellular glutathione (GSH) synthesis. GPx4 uses GSH to detoxify lipid hydroperoxides and plays a key role in preventing ferroptosis by reducing peroxidized phospholipids in cell membranes [15, 16]. As shown in Fig. 4, 14 days of immobilization did not significantly affect SLC7A11/xCT mRNA or GPx4 protein levels (Fig. 4B,C). We next examined superoxide dismutases (SODs), which catalyze the dismutation of superoxide radicals to hydrogen peroxide and oxygen and represent a primary defense against reactive oxygen species. Among the three isoforms, SOD1 and SOD2, the major intracellular enzymes, were not significantly altered, whereas SOD3 expression was markedly increased in the immobilized muscle (Fig. 4D–F). The levels of 4‐HNE, a reactive aldehyde produced by lipid peroxidation that can damage proteins and DNA, were remarkably increased (Fig. 4G). This suggested that casting immobilization led to iron accumulation beyond the buffering capacity of the antioxidant defense system, resulting in enhanced lipid peroxidation.

## Discussion

In this study, we examined the effects of casting‐induced immobilization on nonheme iron accumulation and iron metabolism in skeletal muscles. Fourteen days of immobilization led to an approximately 25% reduction in muscle mass and a significantly increased nonheme iron content in the gastrocnemius muscle. Additionally, disuse atrophy was found to be associated with aberrant upregulation of iron‐uptake and regulatory proteins, along with elevated lipid peroxide levels. The findings suggested that muscle disuse could disrupt iron homeostasis and that iron accumulation might contribute to the pathogenesis of disuse‐induced muscle atrophy.

Iron is essential for numerous cellular processes. However, an excess of it can promote ferroptosis, an iron‐dependent form of regulated cell death characterized by lipid peroxidation. Ferroptosis is increasingly being linked to age‐related diseases. Although iron accumulation in skeletal muscle has been documented in sarcopenia, its involvement in disuse‐induced atrophy remains unclear. In the present study, we observed a significant increase in the levels of both nonheme iron and 4‐HNE, a marker of lipid peroxidation, after 14 days of cast immobilization in 8‐week‐old mice. The results indicated that iron accumulation in skeletal muscle can occur not only as a result of aging but also over a relatively short period due to disuse. Furthermore, our data suggested that disuse rapidly altered the balance between the iron uptake and export systems.

Iron is essential for numerous cellular processes. However, an excess of it can promote ferroptosis, an iron‐dependent form of regulated cell death characterized by lipid peroxidation. Ferroptosis is increasingly being linked to age‐related diseases. Although iron accumulation in skeletal muscle has been documented in sarcopenia, its involvement in disuse‐induced atrophy remains incompletely understood. In this context, Hofer et al. reported that 14 days of hindlimb suspension in rats increased nonheme iron content and oxidative damage in gastrocnemius muscle, particularly in small atrophied fibers, indicating that disuse can disturb iron homeostasis and redox balance in skeletal muscle [17]. In the present study, we observed a significant increase in the levels of both nonheme iron and 4‐HNE, a marker of lipid peroxidation, after 14 days of cast immobilization in 8‐week‐old mice. These results indicate that iron accumulation in skeletal muscle can occur not only as a result of aging but also over a relatively short period due to disuse and that disuse‐induced iron dysregulation is accompanied by enhanced lipid peroxidation. Furthermore, our data suggest that disuse rapidly alters the balance between the iron uptake and export systems, extending previous observations in hindlimb suspension models and implicating dysregulated iron metabolism in the early phase of disuse‐induced muscle atrophy.

Intracellular iron levels are tightly regulated by the iron regulatory protein/iron‐responsive element (IRP/IRE) system. Although both IRP1 and IRP2 participate in this network, they are not functionally equivalent. IRP1 can switch between an RNA‐binding form and a cytosolic aconitase, whereas IRP2 lacks aconitase activity and acts predominantly as a post‐transcriptional regulator of iron metabolism *in vivo* [18]. Genetic ablation studies have further demonstrated that loss of IRP2, rather than IRP1, leads to profound disturbances in systemic iron homeostasis, indicating that IRP2 plays a dominant role in controlling the expression of iron uptake and storage proteins [19]. Based on this evidence, we focused on IRP2 as a key mediator of iron dysregulation in disuse‐induced muscle atrophy. In our model, both IRP2 and transferrin receptor 1 (TfR1) were paradoxically upregulated in immobilized muscles, despite elevated intracellular iron levels. This pattern suggests an impairment of canonical IRP/IRE regulation during disuse. Although the underlying mechanism remains unclear, one plausible explanation is impaired degradation of IRP2. Proteasomal degradation of IRP2 requires heme and iron–sulfur (Fe–S) clusters, which are highly sensitive to oxidative stress and prone to damage [20, 21, 22]. In the present study, there was no decrease in heme‐containing proteins such as myoglobin and cytochrome c in immobilized muscle, suggesting that heme synthesis was not impaired. Therefore, disruption of Fe–S clusters may have prevented IRP2 degradation, leading to its accumulation despite increased intracellular iron. Further studies will be required to clarify the contribution of Fe–S cluster instability to IRP/IRE dysregulation under oxidative conditions and to define the coordinated roles of IRP1 and IRP2 in disuse‐induced muscle atrophy.

GPx4 is a key antioxidant enzyme that detoxifies lipid hydroperoxides into nontoxic lipid alcohols using glutathione as a reducing cofactor, thereby playing a central role in ferroptosis suppression. Notably, overexpression of GPx4 in mice attenuated 4‐HNE accumulation induced by tail suspension [19]. However, previous studies have suggested that GPx4 is not typically upregulated in response to oxidative stress in skeletal muscle [23]. Consistent with this concept, our study found no compensatory increase in GPx4 expression in the immobilized muscles. In addition, the major intracellular superoxide dismutase isoforms, SOD1 and SOD2, were not altered by disuse, whereas only SOD3 showed a significant increase. Because SOD3 is secreted into the extracellular space, this selective upregulation is unlikely to sufficiently counteract intracellular oxidative stress and lipid peroxidation associated with iron accumulation. Thus, the lack of induction of GPx4 and intracellular SOD1–2, together with the limited increase in SOD3, may have contributed to the accumulation of 4‐HNE, indicating that the overall antioxidant defense remains insufficient to mitigate iron overload–induced oxidative stress in disuse muscle.

## Conclusion

This study revealed that casting‐induced muscle atrophy is associated with nonheme iron accumulation, IRP2/TfR1 dysregulation, and lipid peroxidation in skeletal muscles. These findings suggest that oxidative stress‐related iron dysregulation may contribute to early pathological changes in muscle disuse atrophy and might offer potential therapeutic targets for muscle preservation during immobilization.

## Conflict of interest

The authors declare no conflict of interest.

## Author contributions

HY was involved in writing—review and editing, writing—original draft, visualization, validation, investigation, formal analysis, data curation. KH was involved in writing—review and editing, writing—original draft, supervision, project administration, methodology, investigation, funding acquisition, conceptualization. NN was involved in writing—review and editing, supervision, funding acquisition, conceptualization. All authors read and approved the final version of this manuscript.

## Data Availability

All representative data are contained within the article. The data are available from the corresponding author upon reasonable request.
